# Human Lateralization, Maternal Effects and Neurodevelopmental Disorders

**DOI:** 10.3389/fnbeh.2021.668520

**Published:** 2021-03-22

**Authors:** Gianluca Malatesta, Daniele Marzoli, Giulia Prete, Luca Tommasi

**Affiliations:** Department of Psychological, Health and Territorial Sciences, University “G. d’Annunzio” of Chieti and Pescara, Chieti, Italy

**Keywords:** laterality, hemispheric asymmetry, mother-infant interaction, cradling-side bias, behavioral epigenetics, autism spectrum disorders

## Abstract

In humans, behavioral laterality and hemispheric asymmetries are part of a complex biobehavioral system in which genetic factors have been repeatedly proposed as developmental determinants of both phenomena. However, no model solely based on genetic factors has proven conclusive, pushing towards the inclusion of environmental and epigenetic factors into the system. Moreover, it should be pointed out that epigenetic modulation might also account for why certain genes are expressed differently in parents and offspring. Here, we suggest the existence of a sensitive period in early postnatal development, during which the exposure to postural and motor lateral biases, expressed in interactive sensorimotor coordination with the caregiver, canalizes hemispheric lateralization in the “typical” direction. Despite newborns and infants showing their own inherent asymmetries, the canalizing effect of the interactive context owes most to adult caregivers (usually the mother), whose infant-directed lateralized behavior might have been specifically selected for as a population-level trait, functional to confer fitness to offspring. In particular, the case of the left-cradling bias (LCB; i.e., the population-level predisposition of mothers to hold their infants on the left side) represents an instance of behavioral trait exhibiting heritability along the maternal line, although no genetic investigation has been carried out so far. Recent evidence, moreover, seems to suggest that the reduction of this asymmetry is related to several unfavorable conditions, including neurodevelopmental disorders. Future studies are warranted to understand whether and how genetic and epigenetic factors affect the lateralization of early mother-infant interaction and the proneness of the offspring to neurodevelopmental disorders.

## Behavioral Epigenetics and The Development of Lateralization

Studies on lateralization have progressed at a remarkable pace in recent decades, gathering multiple levels belonging to different disciplines and traditions of research. Neural, behavioral and genetic aspects of asymmetries are becoming more and more connected to each other in the all-encompassing framework of biological evolution. Theoretical models suggest that interactive behaviors are key to the evolution of population-level lateral biases (e.g., Ghirlanda and Vallortigara, 2004): a stable equilibrium in the asymmetrical distribution of lateralized behavioral phenotypes of a given species might be reached through the fitness contribution of both antagonistic and synergistic interactions occurring among its members (Ghirlanda et al., 2009). Empirical evidence seems also to suggest that early development is a crucial context in which synergistic interactions affect lateralization (Karenina et al., 2017). However, only rarely evolutionary accounts of lateralization including developmental plasticity as a determining factor have been suggested (e.g., see Michel et al., 2018).

In humans, the ontogeny of lateralization emerges from the multifaceted interaction between genetic and environmental factors that have not been understood in full detail (Güntürkün and Ocklenburg, 2017). Structural asymmetries of the brain are but a small fraction of the *Bauplan* of neural lateralization—the largest part being expressed in the form of functional asymmetries—and they consist in the allocation of different roles to two structurally similar brain hemispheres (Corballis, 2017). Functional asymmetries are ubiquitous in the nervous system especially in the neocortex, and they emerge in many behavioral and mental functions, including action (Guiard, 1987; Serrien and Sovijärvi-Spapé, 2015), imagination (Marzoli et al., 2011a,b, 2013, 2017a; Prete et al., 2016b; Altamura et al., 2020), perception (Marzoli and Tommasi, 2009; Brancucci and Tommasi, 2011; Prete et al., 2015d, 2018b; Prete and Tommasi, 2018), emotion (Prete et al., 2014a, 2015a,c; Wyczesany et al., 2018), attention (Yamaguchi et al., 2000; Chen and Spence, 2017) and memory (Iidaka et al., 2000; Penolazzi et al., 2010; D’Anselmo et al., 2016). Language can be considered the most emblematic case of functional asymmetry, also because the history of discoveries on brain lateralization (and localization) began precisely with aphasia studies (Leblanc, 2017). Nevertheless, it must be noted that motor functions deserve a special place in this list, particularly because of the peculiar status of handedness as a function that is lateralized both behaviorally and neurologically from early childhood (Bondi et al., 2020): around 90% of humans show a preference for using the right hand, which is controlled by the left brain hemisphere (McManus, 2002; Tommasi, 2009). Additionally, footedness should also be granted a special position in the field of human laterality, having been shown to share similarities with handedness both in behavioral and neuropsychological terms, and to be less influenced by cultural and social factors than handedness (Elias and Bryden, 1998; Tran et al., 2014; Packheiser et al., 2020a,c). Population-level motor asymmetries which seem to be precursors of handedness are observed already during fetal life (Hepper et al., 1990; Hepper, 2013; see also Baciadonna et al., 2010 for analogous early predictors of limb laterality in a non-human species), speaking in favor of a substantial genetic contribution. In this regard, the search for genetic factors of human functional lateralization has been characterized by single- or multiple-gene theories aimed to explain handedness, and continues nowadays within molecular genetics studies addressed to the identification of specific loci (Cuellar-Partida et al., 2021). Interestingly, these studies also suggest a partly common ground among genetic variants influencing the development of brain functional laterality and the emergence of neurodevelopmental disorders (Wiberg et al., 2019). However, no evidence has proven strong enough to exactly explain the statistical frequencies of hand preference observed in families (Medland et al., 2009; McManus et al., 2013; Armour et al., 2014). Environmental factors have been therefore implicated, from the effect of hormones (Geschwind and Galaburda, 1985; Berretz et al., 2020) and fetus position *in utero* (Previc, 1991), to the visual experience of own and others’ hands during early infancy (Michel and Harkins, 1986; Fagard and Lemoine, 2006). Michel et al. (2018) suggested that the development of lateralization begins prenatally, and progresses postnatally as a head orientation preference, predominantly right-biased in infants (Michel and Harkins, 1986). Such an early rightward postural asymmetry would have the effect of placing their right hand in their visual field more than their left hand, thus causing cascading feedback-based proprioceptive effects during movement, possibly facilitating the gradual emergence of right-handedness. This suggestion was also confirmed by the observation of children with congenital muscular torticollis, whose restricted early visual experience affected the later development of handedness (Ocklenburg et al., 2010). On the other hand, right-handedness might also be fostered by children imitating adult’s manual preferences (Fagard and Lemoine, 2006). Similar mechanisms might be involved not only in the development of handedness, but also in the attentional bias toward the right side of others’ body observed in both right- and left-handers (Marzoli et al., 2015, 2017a,b, 2019; Lucafò et al., 2016, 2021; see also Marzoli et al., 2014), which in turn could account for the left-handers’ advantage in fighting and sports (e.g., Groothuis et al., 2013). Although the relative weight of genetic and environmental determinants of handedness has not been established yet, epigenetic effects have been hypothesized at both the molecular (Leach et al., 2014) and the behavioral level (Schmitz et al., 2017), and the same should be true for other instances of functional asymmetries.

In addition to prenatal processes occurring *in utero* (e.g., Ocklenburg et al., 2017), behavioral epigenetics could play a major role during postnatal life, specifically because of parental care: humans, as many mammalian species, are indeed characterized by altriciality, that is an extended period after birth during which the newborn is helpless and depends on external sources (i.e., adults) for survival (Gubernick, 2013). This means that the social and behavioral environment is crucial—through an extraordinarily complex matrix of variables—for development. This “epigenetic niche” exerts an effect on the offspring’s endophenotype, bringing about the expression of the genes in an environment shared with the caregivers. Importantly, the social bonding between parent and offspring is an environment in and of itself, and since the attachment behavioral system is the predisposed motivational structure that brings the infant and the mother to seek proximity to each other (Simpson and Belsky, 2008; Norholt, 2020), it may well constitute a very powerful context for the development of laterality. In this frame, lateralization research might take advantage of an important example of epigenetic niche: in the last decades, in fact, “cradling behavior” emerged as a specific case of lateralized social behavior involving parent (in particular the mother) and child, potentially modulating the development of hemispheric lateralization (Packheiser et al., 2019b).

## Cradling-Side Bias as Maternal Effect

**Figure 1:**
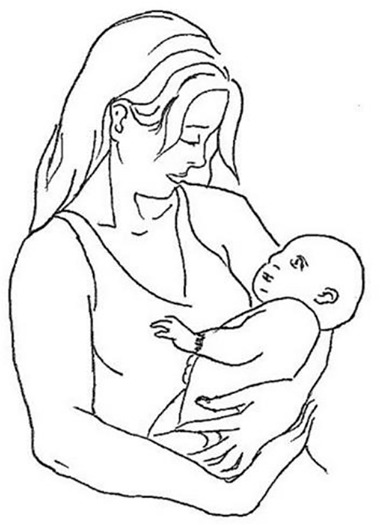
An example of left-cradling bias (LCB).

Cradling behavior has been consistently reported as left-lateralized at the population level, especially in women (65–70% of women cradle infants to the left of their body midline; see Figure 1; Packheiser et al., 2019b), and the bias has been causally linked to the development of the right hemisphere (Manning and Chamberlain, 1991; Harris et al., 2001; Bourne and Todd, 2004).

Indeed, it has been shown that the left-cradling bias (LCB) sets the postural conditions that facilitate an optimal emotional attunement between adult and infant because the right brain hemispheres of both are predominantly engaged during interactions in which the infant is held on the left side of the adult (Harris et al., 2010). This bias can be supposed to provide the infant with what Gilbert Gottlieb called “experiential canalization” (Gottlieb, 1991), a form of supervised narrowing of experience that the infant is predisposed to receive during a precise period. This is supported by a great amount of evidence: (i) in adults, cradling behavior is more strongly left-biased during the first year of life of the child and then declines in strength (Dagenbach et al., 1988); (ii) adults are selectively biased to the left when cradling (or even imagining cradling) infants or dolls rather than when holding or carrying inanimate objects (Harris et al., 2000); (iii) females are significantly more left-biased than males (Packheiser et al., 2019b); and (iv) the LCB seems to be transmitted from mother to daughter as a sex-linked inherited trait (Manning and Denman, 1994). In light of this evidence, it could be argued that the adult genes encode for the presence of an “obligatory” behavior in the mother-infant attachment during a “sensitive period” of the infant’s development, and for a population-level predisposition to implement it asymmetrically on the left side. The experiential side of the story would consist of the interaction and sensorimotor coordination between adults and infants arising from the LCB. From this perspective, such an experience might modulate epigenetically the direction of the development of typical brain lateralization, triggered and scaffolded by the parent or the caregiver. Interestingly, the stronger LCB in females and the related maternal intergenerational transmission might be consistent with epigenetic studies indicating that certain genes are expressed differently in parents and offspring, as occurs in the case of differential parental imprinting (e.g., maternally derived duplications of a specific portion of chromosome 15 lead to an increased risk of autism and schizophrenia more than analogous paternally derived duplications; Cook et al., 1997; Isles et al., 2016).

A further aspect of this epigenetic view is that the LCB could be advantageous from an evolutionary perspective, because it is correlated to fitness-related traits in mothers, and possibly in children. For instance, research has shown that the correlates of cradling are indirectly evident when comparing women showing different degrees of left (typical) or right (atypical) cradling (Malatesta et al., 2019a,b, 2020b), bringing to the hypothesis that an atypical trajectory in maternal cradling might be one early sign of interference of dyadic socio-emotional communication, and thus of potential neurodevelopmental dysfunctions (Malatesta et al., 2020a, d). The fact that this left-sided population-level asymmetry goes in the direction opposite to that of a majority of right-handers, moreover, provides an important hint that it possibly attained a special functional status during evolution, and this speculation is further supported by the presence of an LCB also in left-handers. In this regard, it should be noted that the bias is detectable also in left-handers, indicating that it does not depend upon the fact that holding on the left would free the adult’s dominant hand (Packheiser et al., 2019b). As such, the epigenetic niche represented by the mother cradling the baby would consist, in strictly biological terms, in a genuine maternal effect (Maestripieri and Mateo, 2009). This is supported by evidence of sex- and side-dependent effects of social perception obtained in previous works—for instance, the fact that the well-known left-face bias seems to be stronger for female faces, suggesting a greater sensitivity for the female face in the right hemisphere (Parente and Tommasi, 2008; Prete et al., 2016a, 2017), and the fact that females showing an LCB are more likely attracted by the left rather than right profile of a baby compared to females showing the opposite bias (Malatesta et al., 2020c).

Among the main explanations suggested for the LCB, the right-hemisphere hypothesis—the most accredited one today—revolves around the interaction and the socio-emotional information exchanged between the cradling and the cradled individual (Manning and Chamberlain, 1991; Harris et al., 2001; Bourne and Todd, 2004; for similar considerations in non-human species see Giljov et al., 2018). According to this hypothesis, the right hemisphere should be mainly involved in emotional processing (Levy et al., 1983; Gainotti, 2012; Prete et al., 2014b, 2015b, 2018a), leading to a left hemibody and hemiface superiority in both the expression and the encoding of emotions. Similarly, evidence confirming the right-hemisphere hypothesis has been collected also for other lateralized social behaviors such as embracing and kissing (Ocklenburg et al., 2018; Packheiser et al., 2019a, 2020b). Therefore, cradling might represent a specific interactional framework benefiting both the mother and the infant, whose lateralization has unlikely been left to chance by evolutionary pressures. From the mother’s point of view, the left-side positioning might facilitate the monitoring of her infant’s well-being cues through her left visual and auditory fields, which project more directly to her right hemisphere (i.e., the one more involved in social and emotional processing; Brancucci et al., 2009; Prete et al., 2020a, b). Consistently, left-cradling individuals exhibit a stronger leftward bias for the processing of emotions from faces (Harris et al., 2001, 2010; Bourne and Todd, 2004). Moreover, the discovery of a preference for the left profile of infants in women showing a left-cradling bias (Malatesta et al., 2020c) suggests that a further adaptive function of the LCB might consist in a facilitated monitoring of the left hemiface of the infant, which is considered more expressive (Mendolia and Kleck, 1991) and whose emotional valence is identified more accurately, especially when a negative emotion is displayed (Kleck and Mendolia, 1990). Similarly, the LCB might expose the right hemisphere of children to the more expressive side of the mother’s face (Hendriks et al., 2011). It is also possible to suppose that this double interaction (Table 1) gave an important advantage to both mothers and infants during the evolution by fostering typical neurodevelopment in the cradled infants.

**Table 1:** Table summarizing the double interaction of left-cradling bias (LCB) functions from the perspective of mother and infant.

Mother	Infant
Monitoring the infant through the left visual and auditory fields.	Exposure to the mother’s left-hemiface.
Exposure to the infant’s left-hemiface.	Monitoring the mother through the left visual and auditory fields.

In this regard, it has been shown that individuals cradled on the mother’s right side during infancy showed a significant decrease of the typical left bias for emotional faces compared to left-cradled individuals, suggesting that mothers’ cradling laterality has crucial outcomes on their children’s development of socio-emotional abilities, such as the ability to perceive facial emotions later in life (Vervloed et al., 2011).

## Cradling Behavior and Neurodevelopmental Disorders

The role of the LCB in facilitating emotional communication is supported by findings suggesting that a reduction or inversion of the typical cradling lateralization is associated with several factors that might interfere with the quality of the mother-infant relationship and be a sign of a lack of wellbeing in the cradling woman. In previous studies, we showed that a reduction of the LCB is related to: (i) reduced empathy and increased depressive symptoms in mothers (Malatesta et al., 2019b); (ii) non-optimal patterns of attachment styles in females (Malatesta et al., 2019a); and (iii) prejudiced attitudes towards the cradled individual’s ethnic group in females (Malatesta et al., 2020b). Similarly, the negative association between atypical (right) cradling and the quality of the mother-infant relationship seems to be confirmed by the fact that stress and negative affective states reduce the leftward asymmetry (Bogren, 1984; Weatherill et al., 2004; Suter et al., 2007, 2011; Reissland et al., 2009; Scola et al., 2013; Boulinguez-Ambroise et al., 2020; Pileggi et al., 2020). Furthermore, a link between this population-level bias and the later development of a typical cognitive and socio-emotional functioning has been suggested by recent findings associating developmental disorders—such as autism spectrum disorder (ASD)—and atypical patterns of lateralization in cradling (Jones, 2014; Pileggi et al., 2015; Forrester et al., 2019, 2020; Herdien et al., 2020; Malatesta et al., 2020a, d). This link is also highlighted by evidence unveiling that ASD constitutes a group of neurodevelopmental disorders that, besides entailing chronic and severe impairment in socio-communicative and empathic relationships, are also characterized by an early hypolateralization of brain functions (e.g., Escalante-Mead et al., 2003; Stroganova et al., 2007), including a reduced left bias for faces (Ashwin et al., 2005; Dundas et al., 2012). Furthermore, given that parents of children with ASD exhibit autistic traits to a greater extent compared with controls (Bishop et al., 2004; Ruta et al., 2012; Bora et al., 2017) and given that autistic traits in adults are associated with a reduced LCB (Fleva and Khan, 2015), we have hypothesized an association between reduced left-cradling preference in mothers and later diagnosis of ASD in children (Malatesta et al., 2020a, d). This perspective is in line with research on other forms of systematic deviation from the typical behavioral lateralization such as left-handedness. For example, although the issue is still debated (McManus, 2019), left-handedness has been related to several impairments (e.g., in cognitive abilities such as intelligence and spatial abilities; Gibson, 1973; Johnston et al., 2009; Nicholls et al., 2010; Papadatou-Pastou and Tomprou, 2015; Somers et al., 2015) and has been considered as a cue of reduced fitness (e.g., for evidence in favor of a relation between reduced right-handedness and decreased academic and socioeconomic success see Deary et al., 2007; Strenze, 2007), along with other negative predictors of fitness (e.g., fluctuating asymmetries such as ear, digit, or wrist asymmetries; Manning et al., 1997) which have been related to atypical brain asymmetries (Thoma et al., 2002) and left-handedness itself (Kobyliansky and Micle, 1986).

## Conclusion

We propose the idea that human caregivers play a canalizing role during a sensitive period of developmental plasticity *via* their own lateralized motor patterns. These would give rise in the infant to lateralized experiences in multiple sensory modalities, due to the bidirectional nature of interactive behavior at very close contact. Of all biases, the case of cradling would be extremely interesting to examine with such an approach because its obligatory and simple nature could qualify it as a major epigenetic determinant of neural lateralization. Moreover, the LCB could be the access point to a wider pattern of lateralized adult-infant interactive and social behaviors (embracing, caressing, kissing, cuddling, tickling, whispering, et cetera) acting as epigenetic niches for typical development. Further studies are needed to establish associations among the lateralized experience provided by those interactive behaviors, hemispheric asymmetries, and motor, cognitive and socioemotional development. Given the role of the attachment system as a regulator of proximity seeking (Simpson and Belsky, 2008), and the previous evidence linking the cradling side to attachment in adults (Malatesta et al., 2019a), a major target should be the search for links among the observed patterns of infant attachment and the aforementioned motor, neural and developmental variables. Furthermore, cradling behavior has coevolved with the infant’s proclivity to actively cling onto the caregiver (Berecz et al., 2020), and being held or carried on the left or the right side of the adult’s body imposes complementary degrees of freedom on the infant’s left and right upper limbs. Thus, a direct effect of adult-infant postural laterality is expected to be manifested in the differential use of arms and hands by the infant. More specifically, it is possible to predict that left-sided cradling favors the development of right-handedness in the infant, an effect already assessed in nonhuman primates (Hopkins, 2004) and investigated only partially in humans (Scola and Vauclair, 2010).

Based on the state-of-the-art on the cradling-, embracing- and kissing-side bias research, a better understanding of the adaptive role of these behavioral asymmetries appear desirable to verify their potential function. For example, although research carried out since 1960 has examined the possible correlations between typical/atypical cradling lateralization and several variables in different populations, we do not know much about its association with typical brain organization and increased fitness, and the possible outcomes on the offspring of being cradled on the left or the right during infanthood. Compared to other asymmetrical patterns of brain organization (e.g., handedness), cradling behavior necessarily involves the joint participation of two individuals: one cradling and another being cradled. In this regard, it is plausible that lateral cradling preferences are strongly associated with affective functioning, which is known to be strongly impaired in disorders such as autism, schizophrenia, and alexithymia (Tordjman, 2008).

To conclude, this perspective aims to encourage the detailed study of the nature and effects of the motor and sensory lateral biases expressed in the context of adult-infant interactive behavior. Due to the difficulties in directly manipulating such a dyadic interaction to show possible causal effects in humans, the involvement of animal models might be a useful approach (Manning et al., 1994; Karenina et al., 2017; Giljov et al., 2018; Boulinguez-Ambroise et al., 2020). Moreover, the lateral preference stability over time has received little attention to date, with conflicting findings (Dagenbach et al., 1988; Manning, 1991; Scola et al., 2013; Todd and Banerjee, 2016; Malatesta et al., 2020a). Therefore, the dynamics and spatiotemporal progression of the active and passive biases of the dyad over time should be investigated with a microgenetic approach, and their directionality and strength should be associated with longitudinal assessments of hemispheric asymmetries, cognitive development, and the pattern of attachment between parent and infant.

## Data Availability Statement

The original contributions presented in the study are included in the article, further inquiries can be directed to the corresponding author.

## Author Contributions

All authors listed have made a substantial, direct and intellectual contribution to the work, and approved it for publication.

## Conflict of Interest

The authors declare that the research was conducted in the absence of any commercial or financial relationships that could be construed as a potential conflict of interest.
